# From Excessive Journal Self-Cites to Citation Stacking: Analysis of Journal Self-Citation Kinetics in Search for Journals, Which Boost Their Scientometric Indicators

**DOI:** 10.1371/journal.pone.0153730

**Published:** 2016-04-18

**Authors:** Petr Heneberg

**Affiliations:** Third Faculty of Medicine, Charles University in Prague, Praha, Czech Republic; Universidad de Las Palmas de Gran Canaria, SPAIN

## Abstract

Bibliometric indicators increasingly affect careers, funding, and reputation of individuals, their institutions and journals themselves. In contrast to author self-citations, little is known about kinetics of journal self-citations. Here we hypothesized that they may show a generalizable pattern within particular research fields or across multiple fields. We thus analyzed self-cites to 60 journals from three research fields (multidisciplinary sciences, parasitology, and information science). We also hypothesized that the kinetics of journal self-citations and citations received from other journals of the same publisher may differ from foreign citations. We analyzed the journals published the American Association for the Advancement of Science, Nature Publishing Group, and Editura Academiei Române. We found that although the kinetics of journal self-cites is generally faster compared to foreign cites, it shows some field-specific characteristics. Particularly in information science journals, the initial increase in a share of journal self-citations during post-publication year 0 was completely absent. Self-promoting journal self-citations of top-tier journals have rather indirect but negligible direct effects on bibliometric indicators, affecting just the immediacy index and marginally increasing the impact factor itself as long as the affected journals are well established in their fields. In contrast, other forms of journal self-citations and citation stacking may severely affect the impact factor, or other citation-based indices. We identified here a network consisting of three Romanian physics journals *Proceedings of the Romanian Academy*, *Series A*, *Romanian Journal of Physics*, and *Romanian Reports in Physics*, which displayed low to moderate ratio of journal self-citations, but which multiplied recently their impact factors, and were mutually responsible for 55.9%, 64.7% and 63.3% of citations within the impact factor calculation window to the three journals, respectively. They did not receive nearly any network self-cites prior impact factor calculation window, and their network self-cites decreased sharply after the impact factor calculation window. Journal self-citations and citation stacking requires increased attention and elimination from citation indices.

## Introduction

With the establishment of bibliometric indicators and standards, they started to affect the careers, funding, and reputation of individuals, their institutions and journals themselves. This brought self-citations in all their forms into the spotlight, creating numerous controversies among scholars [1]. Already over a half century ago, Garfield and Sher calculated that 8% of citations were author self-citation, and 20% of citations were journal self-citations [2]. Two types of self-citations were later distinguished, consisting of diachronous (received) and synchronous (made) self-citations [3]. Self-citations share some common patterns, which were fittingly characterized by Glänzel et al. [4] as follows:

1. Self-citations are generally younger and have a shorter half-life than foreign citations
2. Self-citations stabilize in a period of three to four years after publication
3. There is a clear relationship between the number of citations and self-citations received
4. The percentage of self-citations only slightly increases with the number of co-authors.

Journal self-citations show us how often a journal is cited by its own publications [5], which can be used to manipulate the impact factor of journals [6,7]. The journal self-citation data are available from the Journal Citation Reports (JCR) provided as a part of the Web of Knowledge platform by Thomson Reuters. A decade ago, the provider of JCR platform analyzed the journal self-citation ratios for the 5,876 journals in the JCR Science Edition 2002, concluding that 82% of journals had self-citation rates at or below 20%, with a median of just 9%. When the journal self-citations were subtracted from the impact factor for 153 journals in the category Cell Biology, 22 journals showed a change in rank of five or more positions. However, the ranking of top-tier journals was largely unaffected as they received high numbers of self-citations but these were outnumbered by foreign citations [6,8,9]. Importantly, the top-tier journals were recently reported as practicing impact factor boosting by publishing excessive amount of editorial materials (up to 44%), which both cite and are cited by other documents. Combined, the parallel world of documents considered as uncitable by JCR was responsible for up to 30% of the total citations to the top-tier biomedical journals, particularly the *New England Journal of Medicine*, *JAMA* and the *Lancet* [10]. In the lower performing journals, even worse figures are occasionally seen. For example, papers published in the journal *Diagnostica* in 2001 received over 75% of their citations within the window used for impact factor calculation from editorial materials published by the journal itself [11]. Potential manipulation of journal impact factors by journal self-citations has been reported repeatedly by several independent groups [12,13,14,15,16,17,18].

The percentage of author self-citations and foreign citations received displays a specific kinetics. The ageing of author self-citations is much faster than that of foreign citations across probably all science fields [4,19,20]. The author self-citations peak earlier than the foreign citations, and their number is increased with the increasing number of authors of the cited paper [4,19]. A citation window of three post-publication years is considered sufficient for self-citation studies as the author self-citation indicators become relatively stable at post-publication year 3 or 4 [21].

In contrast to author self-citations, little is known about temporal evolution of journal self-citations. Only Rousseau focused on a small set of top-tier and randomly chosen journals, analyzing their self-citation rate within the post-publication years 0, 1 and 10, concluding that the curves of journal self-cites and foreign cites display different kinetics. However, Rousseau analyzed the detailed citation kinetics of only one journal (*Nature*) [22]. In particular, coercive journal self-citations may play a role. These are requested by editors despite the requests a) give no indication that the manuscript was lacking in attribution; b) make no suggestion as to specific articles, authors, or a body of work requiring review; and c) only guide authors to add citations from the editor's journal [23]. In a survey, 6672 respondents commented on their experience with 45,955 papers accepted by 832 journals in the field of economics, sociology, psychology and business. They indicated that 175 of these journals adopted coercive practices, many of them multiple times. The higher ranking journals were found more likely to coerce. And this coercive practice is successful–only 11% of the respondents asked to coerce avoided to do so, and actually most of them added the number of citations requested despite 28% of them were asked to add even five or more citations [23]. There are cases of accusations of coercive practices among journals of similar standing [24], and of course there are denials of such practices by persons connected with journals named as practicing these habits [25]. But we should not blame just the editors. When working as an editor, it is not uncommon to see the peer-reviewers to request the addition of coercive self-citations of the reviewers. There is a good example based on peer-reviews submitted to the *Journal of Psychosomatic Research*, a well-established journal in its field. Of 428 total citations mentioned in the peer-reviews received by this journal in year 2012, 29% were coerced self-cites to the reviewers´ papers. Moreover, the coercive self-cites were twice more common in reviews recommending revision or acceptance (33%) than in those suggesting straight rejection (15%). And whereas only 5% of citations to others´ work were not associated with some rationale, the same was true for 21% of coerced peer-reviewers´ self-cites [26]. Besides that, some other practices may apply. Among the most extreme ones are insertions of citations to the journal (or author) into the articles written by others without their permission, as it was documented in case of Cyril Burt, the past editor of the *British Journal of Statistical Psychology* in 1947–1963 [27]. Clearly, despite it is well known that the journal self-citation practices exist, more details on their kinetics and on reasons leading to their inflation are needed [18].

Here we hypothesized that the kinetics of journal self-citations may show a generalizable pattern within particular research fields or across multiple fields. To answer this hypothesis, we analyzed self-cites to 60 journals from three independent research fields (*Multidisciplinary sciences*, *Parasitology*, and *information sciences*). We also hypothesized that the kinetics of journal self-citations and citations received from other journals of the same publisher may differ from the pattern of foreign citations. To answer this hypothesis, we analyzed the journals published by three publishers, namely *Science Signaling* (published by the American Association for the Advancement of Science), *Nature Immunology* (published by Nature Publishing Group) and *Proceedings of the Romanian Academy*, *Series A* (published by Editura Academiei Române). The first two of them were selected because they adopted the publication model involving publication of numerous editorial materials discussing the research published in these journals or in other journals of the same publisher [10]. The third group of the journals was identified based on the analysis of raw data used to test the first hypothesis due to unusual citation kinetics and suspected citation stacking.

## Materials and Methods

As a data source, we used Thompson Reuters´ Web of Science (WoS) database platform, which contained the following citation databases: Science Citation Index Expanded (1945-present), Social Sciences Citation Index (1977-present), Arts & Humanities Citation Index (1977-present), Conference Proceedings Citation Index-Science & Social Science & Humanities (1990-present), Emerging Sources Citation Index (2015-present), Current Chemical Reactions (1986-present), Index Chemicus (1993-present), KCI-Korean Journal Database (1980-present), and SciELO Citation Index (1997-present).

We searched the 2014 edition of Journal Citation Reports for 20 top-tier journals in the category *Multidisciplinary sciences*, and another 20 top-tier journals in the category *Parasitology*. The journals in each category were sorted according to their most recent impact factor, and the highest ranking 20 journals in each category were used for further analyses. In addition, we searched for 20 journals, which publish papers in the field of *information science*, particularly scientometrics or bibliometrics. In order to retrieve them, we performed the following WoS search TOPIC: ("scientometric*" or "bibliometric*" or "informetric*" or "citation*"), refined by RESEARCH AREAS: (INFORMATION SCIENCE LIBRARY SCIENCE or SCIENCE TECHNOLOGY OTHER TOPICS or COMPUTER SCIENCE OR SOCIAL SCIENCES OTHER TOPICS). The search revealed 16,399 results, which, however, contained still numerous multidisciplinary journals, proceedings and technologically-oriented journals. These were manually removed, and the first 20 journals according to the number of matching papers published were used for the subsequent analyses. The journals analyzed, their ISSN and IF_2014_ are listed in **S1 Table**.

All the searches were performed during February 2016, thus we considered year 2014 as the last complete available dataset allowing the retrieval of the citation data. For all the 60 analyzed journals, we retrieved the following:

- impact factor in 2007–2014,
- impact factor without journal self-cites in 2007–2014,
- total number of citing articles in 2014 to articles published in 2007–2014 in each respective journal, and
- number of journal self-citing articles in 2014 to articles published in 2007–2014.

For *Science Signaling* and *Nature Immunology*, we retrieved the following:

- number of articles published in 2009–2012,
- total annual numbers of citing articles in 2009–2014 to articles published in 2009–2012,
  ○ of that annual numbers of citing articles in:
    ■ *Science Signaling*,
    ■ *Science*,
    ■ *Nature*,
    ■ any of the 33 *Nature series* journals, and
    ■ *Nature Immunology* in 2009–2014 to articles published in 2009–2012.

To analyze the citation stacking by journals published by Editura Academiei Române (*Proceedings of the Romanian Academy*, *Series A*, *Romanian Journal of Physics*, and *Romanian Reports in Physics*), we retrieved the following:

- impact factor in 2007–2014,
- impact factor without journal self-cites in 2007–2014,
- total number of citing articles in 2014 to articles published in 2007–2014 in each respective journal, and
- number of citing articles published in any of the three physical science journals published by Editura Academiei Române (including journal self-citing articles) in 2014 to articles published in 2007–2014.

The analyzed *Science* and *Nature series* journals and those published by Editura Academiei Române, their ISSN and IF_2014_ are listed in **S1 Table**.

As an “article” we understood any type of the paper listed in the WoS database. For some journals, the impact factors and citations were available only for a part of analyzed period, depending on their coverage by WoS. Although the WoS database contains errors, the retrieved documents were not treated for errors, as it was assumed that the error rate would be similar across the examined time period and document types and thus should not affect the outcomes of this report. As a “network” we understood the group of journals characterized by disproportionally high amount of citations received from journals within the network when compared to the other journals within the same research field. The network of journals may typically consist of journals published by the same publisher, as we revealed for the three physics journals published by Editura Academiei Române. However, such network may also be formed by other mechanisms, typically by the inclusion of identical persons within editorial boards of journals published by multiple publishers [28], by personal relationships between the editors, or other mechanisms, which are beyond the scope of this study. As “network self-cites” we understood any citations received by the analyzed journal from other journals within the “network”. As a “skewed pattern of cites by self-cites” we understood the citation ratios, which were strongly affected, i.e., increased, by the presence of journal self-cites. We quantified the share of variance attributed to these patterns in the Results. As “citation stacking” we understood the behaviour of a network of journals, which work apparently together to cite each other and thus raise their impact factors.

The data were shown as means ± SD unless stated otherwise. Weighted means were calculated for relative self-citation rates, when the means of the citation rates of multiple journals were compared, with the citation rates received by each journal considered equal to each other irrespectively of the total number of citations received by the respective journal. Statistical analyses were performed in PAST version 2.14, and included one-way ANOVA with Tukey’s pairwise comparisons, Levene´s test for homogeneity of variance based on means and medians, Welch F test calculated instead of ANOVA in the case of unequal variances, and correspondence analyses.

## Results

### General Kinetics of Journal Self-Cites

**Fig 1:**
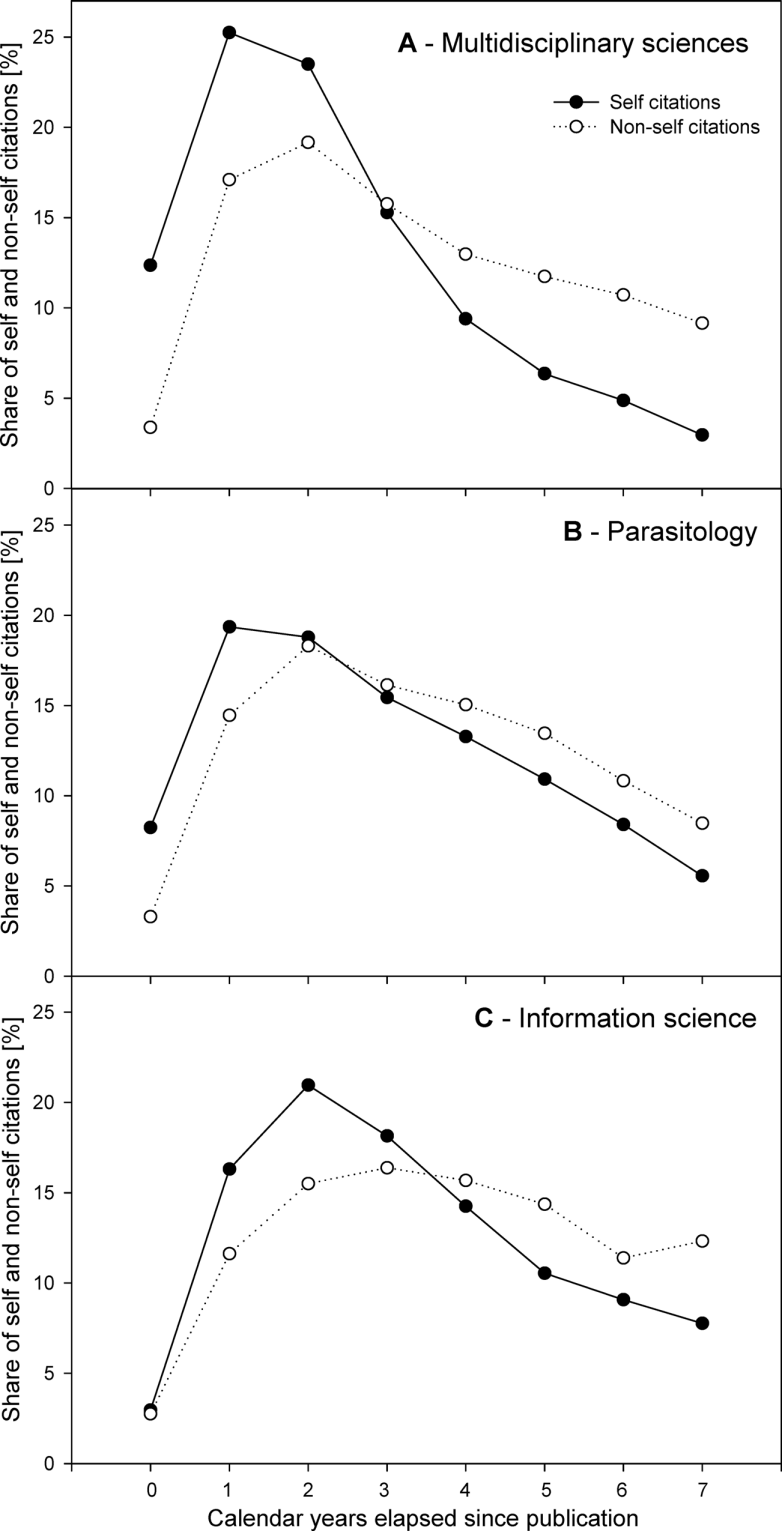
Kinetics of journal self-cites and foreign cites. (**A**)–Top 20 journals from the JCR category *Multidisciplinary sciences* as sorted by IF_2014_, (**B**)–Top 20 journals from the JCR category *Parasitology* as sorted by IF_2014_, (**C**)–Top 20 WoS-indexed journals publishing the articles on *information science* sorted by the number of articles published in the selected research field. Shown are annual shares of journal self-cites (black circles) and non-self, i.e., foreign, cites (white circles). The annual shares were calculated by dividing annual number of self-cites or foreign cites by the total number of self-cites or foreign cites received during post-publication years 0–7.

**Fig 2:**
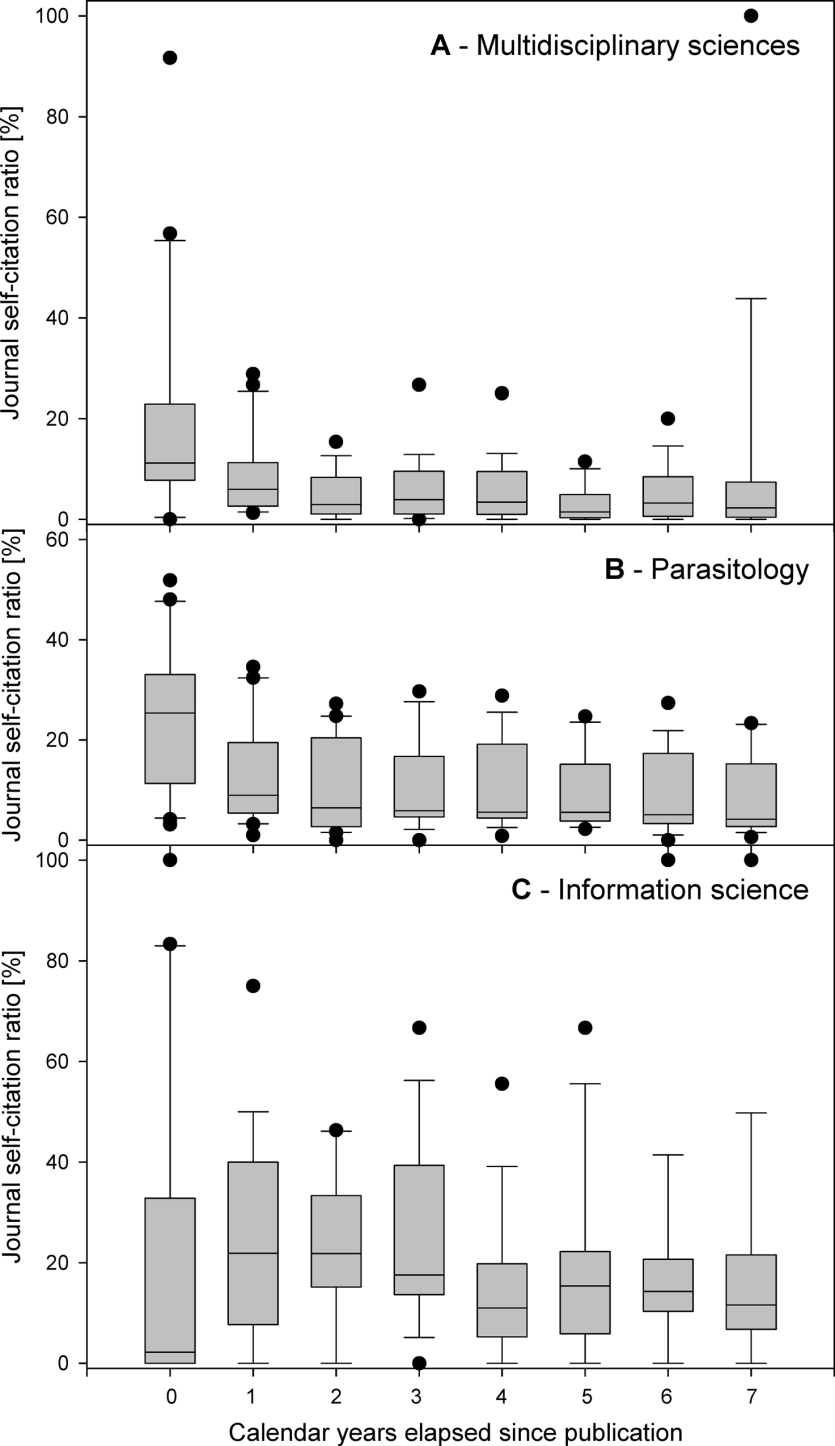
Share of journal self-cites among total cites. (**A**)–Top 20 journals from the JCR category *Multidisciplinary sciences* as sorted by IF_2014_, (**B**)–Top 20 journals from the JCR category *Parasitology* as sorted by IF_2014_, (**C**)**–**Top 20 WoS-indexed journals publishing the articles on *information science* sorted by the number of articles published in the selected research field. Shown are changes in the ratio of self-cites to total cites received in the respective years, analyzed for post-publication years 0–7. Indicated are means, SDs, 95%CIs, and outliers outside of the 95%CI interval.

The WoS category *Multidisciplinary sciences* included 57 journals, with a median IF_2014_ 0.7, aggregate IF_2014_ 5.3 (4^th^ highest among WoS categories), and both aggregate cited and citing half-lives equal to 7.5 years. The top 20 multidisciplinary journals analyzed, sorted by IF_2014_, ranged from *Nature* (IF_2014_ 41.5) to *Scientific American* (IF_2014_ 1.1). The journal self-citations (**Fig 1**) peaked during the post-publication year 1 (13,638 self-cites, 25.3% of self-cites received in post-publication years 0–7) and 2 (12,697 self-cites, 23.5%) and were subject to sharp decrease with the next years, with only 1,601 journal self-cites received during the post-publication year 7 (3.0%). The decrease of a total number of journal self-cites reached 35.0% and 38.4% annually in post-publication years 3 and 4, and remained high at 23.2–39.3% in post-publication years 5–7 (**Fig 1A, black circles**). The kinetics of total cites to the journal was slightly delayed compared to the above, and peaked within the post-publication year 2, when it reached 217,821 cites (19.2% of cites received in post-publication years 0–7). The subsequent decrease was just much less pronounced (**Fig 1A, white circles**), as the foreign cites decreased only by 8.3–16.7% annually. As the above suggested that the journal self-citation ratio is subject to temporal variation, we next analyzed its kinetics in time during post-publication years 0–7 (**Fig 2**). Despite each journal displayed somewhat different kinetics, the general self-citation ratio pattern was shared by all the *Multidisciplinary sciences* journals. This pattern consisted of the high self-citation ratio experienced during the post-publication year 0 (mean 14.8; weighted mean 19.9 ± 22.2%, min 0.0, max 91.7), followed by a decrease during year 1 (mean 6.6; weighted mean 8.0 ± 7.7%, min 1.3, max 28.8) and a plateau phase during years 2–7 (means decreasing gradually from 5.5% to 1.5%; weighted means fluctuating among 3.0–9.8%) (**Fig 2A**). Mean effects of self-cites on immediacy index and impact factor are summarized in **Table 1**.

**Table 1:** Effects of self-cites on immediacy index and impact factor calculation. The following journals were analyzed: a) *Multidisciplinary sciences*: 20 journals with the highest IF_2014_ in the JCR category *Multidisciplinary sciences*; b) *Parasitology*: 20 journals with the highest IF_2014_ in the JCR category *Parasitology*; *Information science*: 20 journals with the highest number of papers published on scientometrics or bibliometrics. Weighted means ± SD and ranges are provided for the shares of self-cites.

Category:	*Multidisciplinary sciences*	*Parasitology*	*Information science*
Immediacy index (JCR 2014):			
Mean ± SD	1.387 ± 2.485	0.793 ± 0.680	0.161 ± 0.189
Range	0.102–9.585	0.082–3.148	0.000–0.650
Share of self-cites ± SD [%]	19.9 ± 22.2	24.8 ± 14.6	22.9 ± 32.4
Range of share of self-cites [%]	0.0–91.7	3.1–51.9	0.0–100.0
Impact factor (JCR 2014):			
Mean ± SD	6.818 ± 10.933	3.663 ±2.655	0.989 ± 0.901
Range	1.070–41.456	1.592–12.328	0.104–3.504
Share of self-cites ± SD [%]	5.8 ± 4.9	11.1 ± 9.9	24.1 ± 14.7
Range of share of self-cites [%]	0.0–18.1	0.0–30.3	0.0–57.1

The WoS category *Parasitology* included 36 journals, with a median IF_2014_ 1.6, aggregate IF_2014_ 3.2, aggregate cited half-life 5.3 years, and aggregate citing half-life 8.1 years. The top 20 parasitological journals analyzed, sorted by IF_2014_, ranged from *Cell Host & Microbe* (IF_2014_ 12.3) to *Memórias do Instituto Oswaldo Cruz* (IF_2014_ 1.6). The journal self-citations peaked during the post-publication year 1 (2,096 self-cites, 19.4% of self-cites received in post-publication years 0–7) and 2 (2,034 self-cites, 18.8%) and were subject to a decrease with the next years, with only 602 journal self-cites received during the post-publication year 7 (5.6%). The decrease of a total number of journal self-cites reached 17.7% and 14.4% annually in post-publication years 3 and 4, and intensified in the later years, reaching 17.8%, 23.0% and 33.8% in post-publication years 5, 6 and 7, respectively (**Fig 1B, black circles**). The kinetics of total cites to the journal was slightly delayed compared to the above, and peaked within the post-publication year 2, when it reached 11,570 cites (18.3% of cites received in post-publication years 0–7). The subsequent decrease was just much less pronounced (**Fig 1B, white circles**), as the foreign cites decreased only by 5.4–10.5% annually in post-publication years 3–5, with the decrease intensified to 19.1–19.9% annually in post-publication years 6–7. The general self-citation ratio pattern of the *Parasitology* journals consisted of the high self-citation ratio experienced during the post-publication year 0 (mean 30.0; weighted mean 24.8 ± 14.6%, min 3.1, max 51.9), followed by a decrease during year 1 (mean 18.7; weighted mean 13.0 ± 10.5%, min 1.0, max 34.6) and a plateau phase during years 2–7 (means decreasing gradually from 15.0% to 10.1%; weighted means fluctuating among 8.0–10.6%) (**Fig 2B**). Mean effects of self-cites on immediacy index and impact factor are summarized in **Table 1**.

The journals in the field of *information science* do not aggregate in a single WoS category. Thus, they were searched manually, and, when sorted by IF_2014_, they ranged from *Journal of the American Medical Informatics Association* (IF_2014_ 3.5) to *Investigacion Bibliotecologica* (IF_2014_ 0.1), with one journal lacking its IF due to recent title change (*Journal of the Association for Information Science and Technology*). The journal self-citations peaked during the post-publication year 2 (672 self-cites, 21.0% of self-cites received in post-publication years 0–7) and 3 (582 self-cites, 18.2%) and were subject to a decrease with the next years, with only 249 journal self-cites received during the post-publication year 7 (7.8%). The decrease of a total number of journal self-cites reached 21.5% and 26.0% annually in post-publication years 4 and 5, but later the decrease was only moderate at 13.9% and 14.4% annually in post-publication years 6 and 7, respectively (**Fig 1C, black circles**). The kinetics of total cites to the journal was different, the foreign cites showed a plateau from the post-publication year 2 till the end of the analyzed period, oscillating between 1003 and 1325 foreign cites annually, with the maximum received in post-publication year 4 (16.4% of cites received in post-publication years 0–7) (**Fig 1C, white circles**). The general self-citation ratio pattern of the *information science* journals did not display the high self-citation ratio during the post-publication year 0. Instead, they peaked in post-publication years 1–2 and remained high for the rest of the analyzed period. In post-publication year 0, the self-citation ratio reached mean 23.3% (weighted mean 22.9 ± 32.4%, min 0.0, max 100.0), in year 1 the ratio increased to a mean of 23.3% (weighted mean 26.7 ± 19.6%, min 0.0, max 75.0), and in year 2 the ratio remained high at 27.6% (weighted mean 22.6 ± 14.1%, min 0.0, max 46.3). During post-publication years 3–7, the mean decreased nearly gradually from 23.8% to 15.1% (with weighted means fluctuating among 15.5–25.2%, with very high SDs and journals showing 100% self-citation ratio at years 6–7) (**Fig 2C**). Mean effects of self-cites on immediacy index and impact factor are summarized in **Table 1**.

### The Case of Journals of the *Science* and *Nature* Series

**Fig 3:**
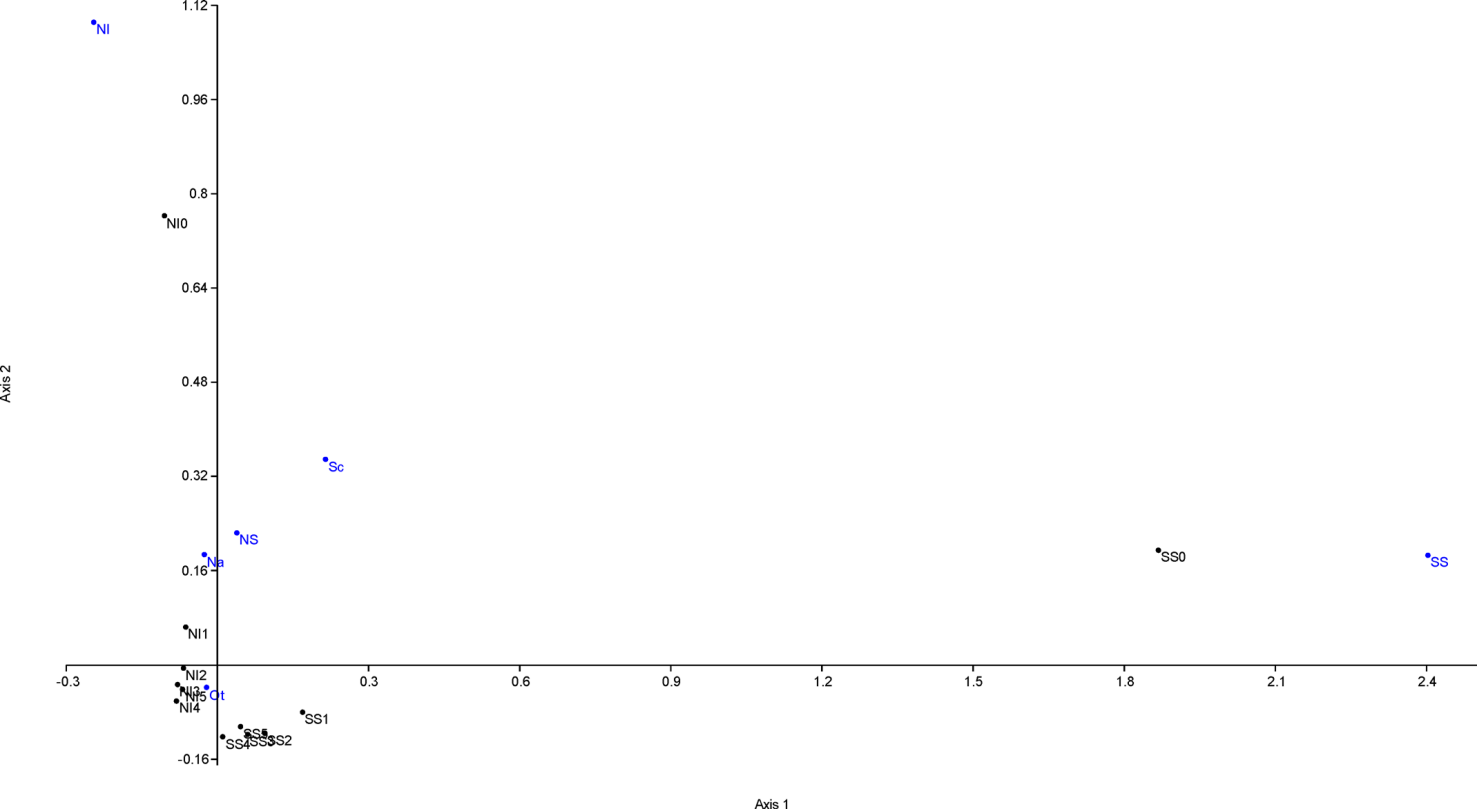
Correspondence analysis (Benzecri scaling) of citations to articles published in *Science Signaling* or *Nature Immunology*. Shown are annual numbers of citations received in post-publication years 0–4 by the two journals (black dots) associated with their source journals (blue dots). The analysis involved cites received in 2009–2014 to articles published in 2009–2012. The resulting factor scores of correspondence analysis are provided within the text of the Results chapter. Abbreviations used–SS: *Science Signaling*, Sc: *Science*, Na: *Nature*, NS: any of the 33 *Nature series* journals, NI: *Nature Immunology*, and Ot: other journals. The citations to the journal are identified by an abbreviation of the journal (SS or NI) together with an indication of the respective post-publication year (0–4).

We next analyzed the citation network of two journals, which rank high among their fields but are not considered the best among the portfolio of their publishers, namely *Science Signaling* (published by the American Association for the Advancement of Science) and *Nature Immunology* (published by Nature Publishing Group). The correspondence analysis of the citations received by articles published by these two journals revealed that the pattern of citations in post-publication years 2–4 are marginally affected by journal self-cites, whereas citations received during post-publication year 1, and especially those received during post-publication year 0 were strongly skewed by journal self-citations. The first ordination axis, which explained 65.6% of variance in the data was highly correlated with self-cites from *Science Signaling* and cites received by articles published in the same journal during the post-publication year 0. The second ordination axis, which explained 33.3% of variance in the data was highly correlated with self-cites from *Nature Immunology* and cites received by articles published in the same journal during the post-publication year 0. Also the number of cites received from *Science* was somewhat higher in both journals, whereas *Nature* and *Nature series* journals were positively associated with early cites to *Nature Immunology* but not *Science Signaling*. Citations from other foreign journals did not display any difference in their kinetics between the two journals or among the years analyzed. (**Fig 3**).

Both, *Science Signaling* and *Nature Immunology* received citations with unequal variance as revealed by Levene's tests for homogeneity of variance based on means (both *p* < 0.001). Thus, we employed Welch F test to analyze the overall differences among the citations received. For *Science Signaling*, the Welch F test revealed significant differences (*p =* 0.01, df = 11.63, F = 5.41). The Tukey's pairwise comparisons suggested that the annual numbers of journal self-cites differed from those received from *Science* (*p* < 0.05, Q = 4.166), *Nature* (*p* < 0.05, Q = 4.227), *Nature Immunology* (*p* < 0.05, Q = 4.190) but not *Nature* series journals (*p* > 0.05, Q = 2.601). The annual numbers of citations received from *Science* did not differ from those received from *Nature* or *Nature Immunology*, and also those received from *Nature* did not differ from those from *Nature Immunology*. However, there were highly significant differences between the annual numbers of citations received from *Nature* series journals when compared either with *Nature*, *Science* or *Nature Immunology* (*p* < 0.001, Q = 6.767; *p* < 0.001, Q = 6.828; *p* < 0.001, Q = 6.791, respectively).

For *Nature Immunology*, the Welch F test revealed significant differences (*p =* 0.002, df = 11.80, F = 8.60). The Tukey's pairwise comparisons suggested that the annual numbers of journal self-cites differed only from those received from *Nature series* journals (*p* = 0.006, Q = 5.482) but not from *Nature*, *Science* or *Science Signaling* (*p* > 0.05 each). There were also no differences in the annual numbers of cites received from *Nature*, *Science* or *Science Signaling* (*p* > 0.05 each). However, the annual numbers of cites received from *Nature series* journals differed from *Nature*, *Science* and *Science Signaling* (*p <* 0.001 each, Q = 9.118, 9.324 and 9.547, respectively).

**Fig 4:**
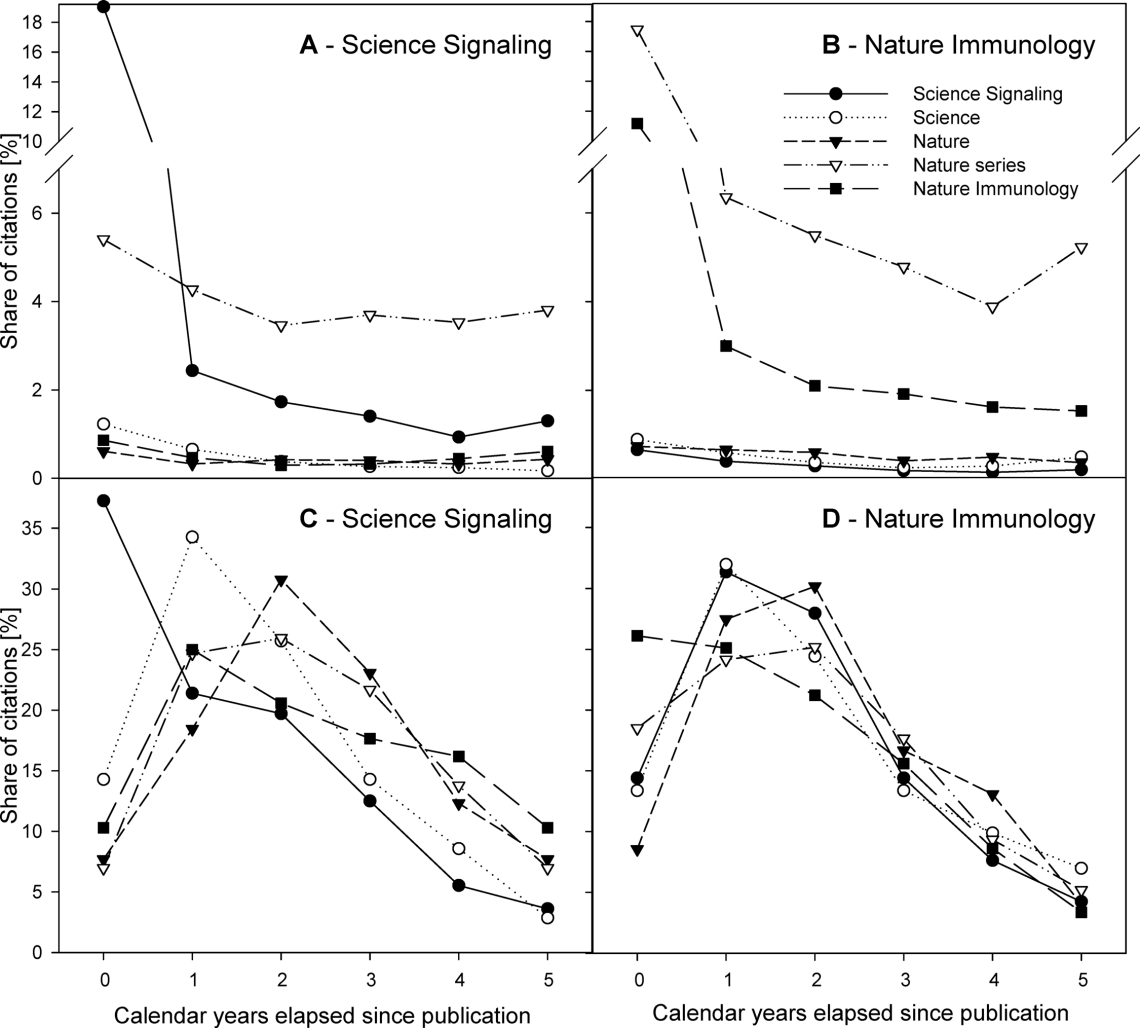
Kinetics of *Science Signaling* and *Nature Immunology* citations. (**A, C**) Cites to *Science Signaling*. (**B, D**) Cites to *Nature Immunology*. (**A, B**) Share of journal self-cites among total cites. Shown are changes in the ratio of cites by the respective journal to total cites received in the respective years, analyzed for post-publication years 0–7. (**C, D**) Kinetics of journal self-cites and foreign cites. The annual shares were calculated by dividing annual number of cites by the respective journal cites by the total number of cites by the respective journal received during post-publication years 0–7. Shown are annual shares of cites by *Science Signaling* (black circles), *Science* (white circles), *Nature* (black triangles), *Nature series* journals (white triangles) and *Nature Immunology* (black squares).

The journal self-citations to *Science Signaling* (**Fig 4**) peaked already during the post-publication year 1 (155 self-cites, 37.3% of journal self-cites received in post-publication years 0–5), and decreased to just 89 (21.4%) and 82 (19.7%) in post-publication years 1 and 2. The sharp decrease continued in later years, with the annual number of journal self-cites reaching 52 (12.5%), 23 (5.5%) and just 15 (3.6%) in post-publication years 3, 4 and 5, respectively. The decrease of a total number of journal self-cites reached 42.6% during post-publication year 1, then it remained at moderate 7.9% during post-publication year 2, and increased to 34.8–55.8% in post-publication years 3–5 (**Fig 4C, black circles**). The kinetics of foreign cites to the journal did not show such pattern even when the top-tier journal of the same publisher was analyzed separately (**Fig 4C, white circles**); similar data were received when analyzing the share of citations received from *Nature*, *Nature Immunology* and any *Nature series* journals. In all these cases, the share of citations received during the post-publication year 0 was low, among 7.0–14.3% but increased to 18.5–34.3% during post-publication year 1, with citations from *Nature* and *Nature series* journals continuing in their rise also during post-publication year 2. In later years, the share of citations received from any of these journals decreased similarly as the journal self-cites did (**Fig 4C**). The above differences were much more prominent when the data were plotted as a ratio of citations received from the respective journal relative to the total number of citations received during the given post-publication year. The ratios of citations received from any of the analyzed journals but *Science Signaling* displayed mutually similar kinetics with slightly higher values during post-publication year 0, and a plateau during the next years analyzed. In contrast, the ratio of journals self-cites reached 19.0% during the post-publication year 0, whereas it dropped to just 2.4% during post-publication year 1, and was even lower (1.7–0.9%) in later post-publication years analyzed (**Fig 4A**).

The journal self-citations to *Nature Immunology* peaked also already during the post-publication year 1 (288 self-cites, 26.1% of journal self-cites received in post-publication years 0–5), but displayed only mild decrease during post-publication years 1–3, when they reached 277 (25.1%), 234 (21.2%) and 172 (15.6%) journal self-cites, respectively. Only later they dropped sharply to 95 (8.6%) and just 37 (3.4%) in post-publication years 4 and 5, respectively. The decrease of a total number of journal self-cites reached just 3.8% during post-publication year 1, then it gradually increased to moderate 15.5% during post-publication year 2, and 26.5%, 44.8% and even 61.1% in post-publication years 3, 4 and 5, respectively (**Fig 4D, black squares**). The kinetics of foreign cites to the journal did not show such pattern with the exception of *Nature series* journals (**Fig 4D, white triangles**); similar data were received when analyzing the share of citations received from *Nature*, *Science* and *Science Signaling*. In all these cases, the share of citations received during the post-publication year 0 was low, among 8.6–14.4% (*Nature series* was at 18.5%) but increased to 27.5–32.0% during post-publication year 1 (*Nature series* increased only moderately to 24.1%), with citations from *Nature* (and *Nature series*) journals continuing in their rise also during post-publication year 2. In later years, the share of citations received from any of these journals decreased similarly as did the journal self-cites (**Fig 4D**). When the data were plotted as a ratio of citations received from the respective journal relative to the total number of citations received during the given post-publication year, the ratios of citations received from any of the analyzed journals but *Nature Immunology* and *Nature series* journals displayed mutually similar kinetics with slightly higher values during first post-publication years, and a plateau during the next years analyzed. In contrast, the ratio of journal self-cites reached 11.2% during the post-publication year 0, whereas it dropped to just 3.0% during post-publication year 1, and decreased further to 1.5–2.1%) during later post-publication years analyzed. Similarly, the ratio of cites received from *Nature series* journals reached 17.5% during the post-publication year 0, whereas it dropped to just 6.4% during post-publication year 1, and remained even lower (at 3.9–5.5%) during later post-publication years analyzed. (**Fig 4B**).

### Citation Stacking by Editura Academiei Române Journals

**Fig 5:**
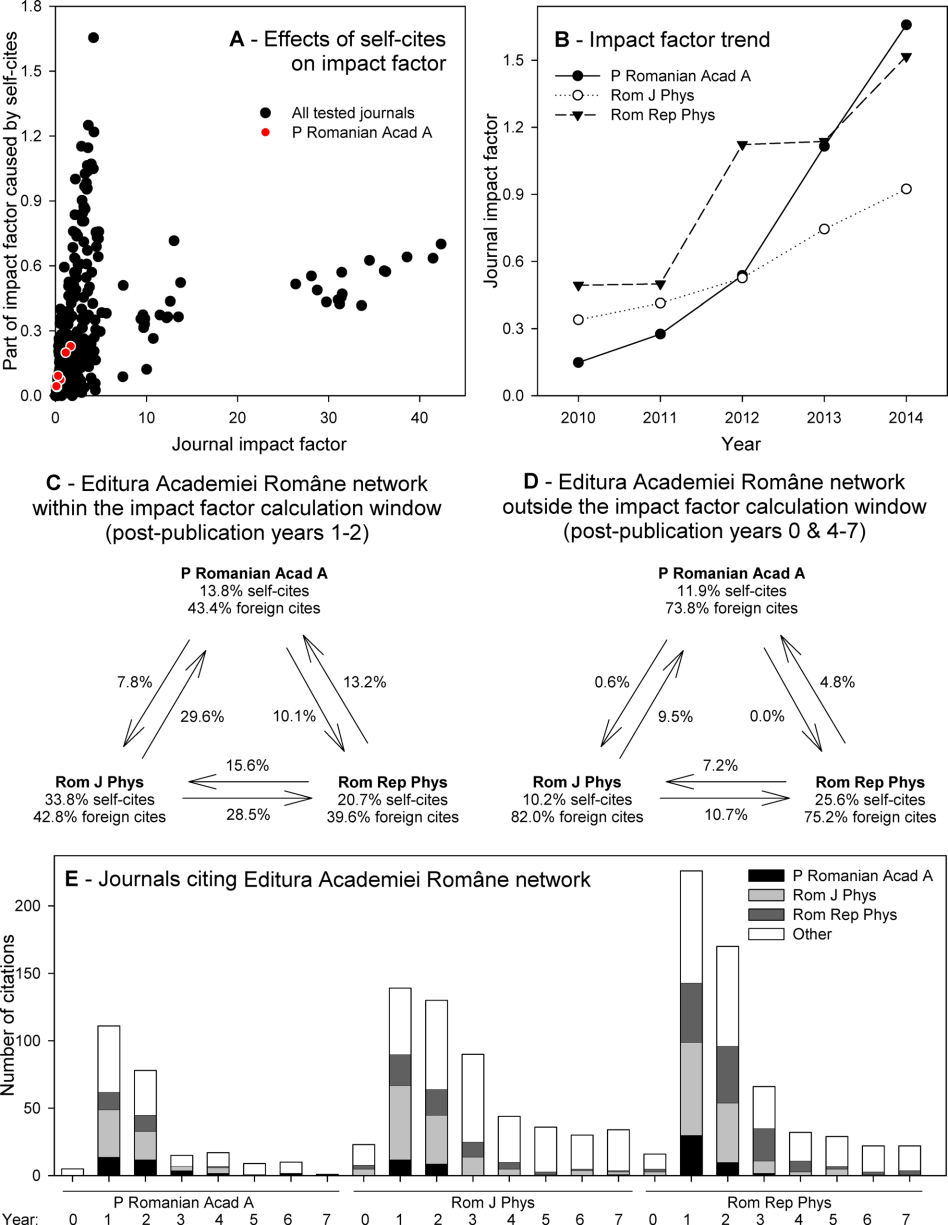
Kinetics of citations to articles in three Romanian physics journals published by Editura Academiei Române. (**A**) Journal impact factor plotted against the part of impact factor caused by self-cites, analyzed individually for the 60 journals examined in course of this study in each year for which the JCR data were available in 2007–2014 (black circles), with highlighted position of *Proceedings of the Romanian Academy*, *Series A* (red dots). (**B**) Changes in JCR impact factor of the three journals published by Editura Academiei Române. (**C-D**) Scheme of the Editura Academiei Române network within the impact factor calculation window, i.e., in post-publication years 1–2 (**C**) and outside the impact factor calculation window, i.e., in post-publication years 0 and 4–7 (**D**). (**E**) Annual number of citations received by the three physics journals published by Editura Academiei Române from their network, matched with foreign citations.

We next analyzed the citation network of three Romanian physics journals published by Editura Academiei Române (**Fig 5**). We identified one of these journals, *Proceedings of the Romanian Academy*, *Series A* (further termed *PRA*), based on the analysis of raw data of *Multidisciplinary sciences* journals, in which it was also listed, as it displayed unusual citation kinetics and suspected citation stacking. Instead of high journal self-citation rate (**Fig 5A**), it displayed high numbers of cites by other journals of the same publisher, namely *Romanian Journal of Physics* (further termed *RJP*) and *Romanian Reports in Physics* (further termed *RRP*). *PRA* experienced recently a dramatic upwards shift of its IF and JCR ranking, being positioned in 4^th^ quartile (46^th^ out of 50 journals) among *Multidisciplinary sciences* journals in 2009, and gradually shifting up to 2^nd^ quartile (15^th^ out of 57 journals) in the most recent JCR edition 2014. Within the same period of time, its impact factor increased from just 0.088 to 1.658 (**Fig 5B**), and also the impact factor without journal self-cites gradually multiplied from 0.044 to 1.429, with all articles published in citable items and with only marginal change in the total number of articles published (39 in 2009, and 49 in 2014). Whereas the citing half-life of articles published in this journal was 7.8 years, the cited half-life was just 2.2 years according to the JCR edition 2014.

Similar changes were observed when analyzing the other two journals. Namely *RJP* recently multiplied its IF and increased JCR ranking, being positioned just in 4^th^ quartile (64^th^ out of 71 journals) among *Multidisciplinary physics* journals in 2009, and gradually shifting up to 3^rd^ quartile (53^th^ out of 78 journals) in the most recent JCR edition 2014. Within the same period of time, its impact factor increased from 0.279 to 0.924 (**Fig 5B**), and also the impact factor without journal self-cites gradually multiplied from 0.205 to 0.611, with all articles published in citable items and with only marginal change in the total number of articles published (100 in 2009, and 103 in 2014). Whereas the citing half-life of articles published in this journal was 7.7 years, the cited half-life was just 3.1 years according to the JCR edition 2014, and with even more striking difference experienced in previous years.

The last of the journals analyzed, *RRP* recently multiplied its IF and increased JCR ranking, being positioned just in 4^th^ quartile (59^th^ out of 71 journals) among *Multidisciplinary physics* journals in 2009, and gradually shifting up to 2^nd^ quartile (32^nd^ out of 78 journals) in the JCR edition 2014. Within the same period of time, its impact factor increased from 0.458 to 1.517 (**Fig 5B**), and also the impact factor without journal self-cites gradually multiplied from 0.379 to 1.187, particularly during the most recent two years. Nearly all articles (94.1–100.0% annually) were published in citable items; the total number of articles published doubled from 68 in 2009 to 113 in 2014. Whereas the citing half-life of articles published in this journal was 9.8 years, the cited half-life was just 2.5 years according to the JCR edition 2014.

**Fig 6:**
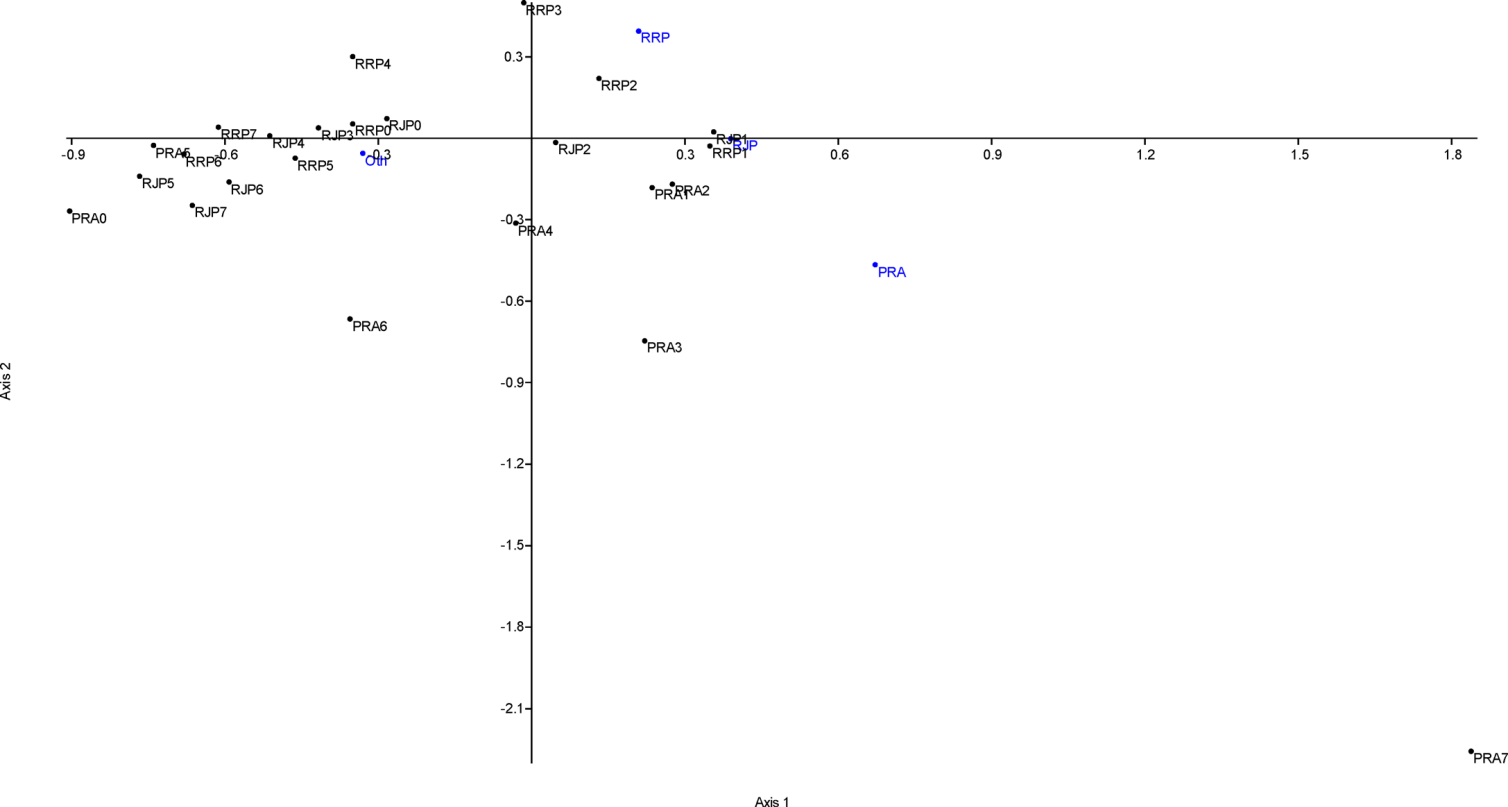
Correspondence analysis (Benzecri scaling) of citations to articles in three Romanian physics journals published by Editura Academiei Române. Shown are annual numbers of citations received in post-publication years 0–7 by the three journals (black dots) associated with their source journals (blue dots). The analysis involved cites received in 2014 to articles published in 2007–2014. The resulting factor scores of correspondence analysis are provided within the text of the Results chapter. Abbreviations used–PRA: *Proceedings of the Romanian Academy*, *Series A*, RJP: *Romanian Journal of Physics*, RRP: *Romanian Reports in Physics*, Oth: other journals. The citations to the journal are identified by an abbreviation of the journal (PRA, RJP or RRP) together with an indication of the respective post-publication year (0–7).

The correspondence analysis of the citations received by articles published by these three journals (**Fig 6**) revealed that the pattern of citations in post-publication years 1–2 are strongly affected by cites from the journals within the network, whereas citations received during post-publications year 0 and 4–7 (i.e., outside the window used to calculate IF) were negatively correlated with the cites from any of the journals within the network (cf. also **Fig 5C and 5D**). The first ordination axis, which explained 68.6% of variance in the data was positively correlated with cites from all three journals within the network, and was negatively correlated with citations from foreign journals. The second ordination axis, which explained 21.9% of variance in the data was correlated with differences in a number of citation received from *RRP* (positive correlation) and *PRA* (negative correlation). Only the cites received in post-publication years 1–2 by any of the three journals, and some cites from *PRA* (years 3 and 7) were positively correlated with axis 1. Post-publication year 4 cites to *PRA* and post-publication year 3 cites to *RRP* were neutral in respect to axis 1, whereas all other cites displayed negative association with axis 1 similarly as cites from foreign journals did (**Fig 6**).

The network of Editura Academiei Române journals received citations with unequal variance as revealed by Levene's tests for homogeneity of variance based on means (both *p* < 0.01). Thus, we employed Welch F test to analyze the overall differences among the annual numbers of citations received, which revealed significant differences (*p <* 0.001, df = 46.54, F = 9.74). The Tukey's pairwise comparisons suggested that the annual numbers of self-cites to the network did not differ among the three journals analyzed (p > 0.05 each), whereas the pattern of cites from foreign journals to the network differed when compared to the pattern received from any of the three journals (*p* < 0.001, Q = 7.479 for *PRA*; *p* = 0.006, Q = 4.805 for *RJP*; and *p* < 0.001, Q = 6.027 for *RRP*).

The self-cites to the network (**Fig 5E**) were at a limit of zero during post-publication year 0 but peaked already during the post-publication year 1 (62 (55.9%) network self-cites to *PRA*, 90 (64.7%) network self-cites to *RJP*, and 143 (63.3%) network self-cites to *RRP*). During post-publication year 2, the number of network self-cites decreased but remained still high at 57.7% of self-cites to *PRA*, 49.2% to *RJP* and 56.5% to *RRP*. In later years, the number of self-cites decreased sharply. During post-publication year 4, *PRA* received just a single network self-cite (11.1% of total cites received during the respective year), *RJP* received three network self-cites (8.3%), and *RRP* received seven network self-cites (24.1%) (**Fig 6E**). Thus, the network self-cites to *PRA* decreased by 84.4% during post-publication year 3, the network self-cites to *RJP* decreased by 60.9–70.0% annually during post-publication years 3–5, and the network self-cites to *RRP* decreased by 63.5% and 68.6% during post-publication years 3 and 4 (**Fig 6E**).

## Discussion

In order to facilitate valuable publications rather than only to inflate publication counts, policy makers have emphasized the impact of publications, which is mostly implemented by evaluation one or more citation indices. In many cases, the situation reached another extreme point: “where a scientist publishes has become much more important than what he is publishing” [29,30]. Such pressure is associated with an increased tendency to various types of unethical behavior, including inflated self-cites. Revealing the depth of the problem in a transparent way and identifying interventions to diminish is thus of public interest [18]. As pointed by McVeigh [8], a relatively high self-citation rate can be due to several factors related to a novelty of a topics or a narrow specialization of the journal on some topic, for which it provides a unique publication venue. It also may simply have a few incoming citations from other sources, whereas it is well recognized among its readers; this could particularly apply for journals, which publish their articles in country-specific languages, as the language barrier significantly limits the spread of such information to readers from other regions. Last but not least, journal self-citation rate may also be affected by sociological factors, as researchers (including myself) cite journals of which they are most aware, which is, however, roughly the same population of journals to which they consider to send their own papers for review and subsequent publication. Despite all these reasons would be relevant, and stay behind a significant part of journal self-citations, there appears to be another reason leading to the increased number of journal self-citations–the editorial practice of the journal [8]. In this regard, of note are the policies of major publishers, such as Elsevier, who state explicitly that “an editor should never conduct any practice that obliges authors to cite his or her journal either as an implied or explicit condition of acceptance for publication… Editors should direct authors to relevant literature as part of the peer review process; however, this should never extend to blanket instructions to cite individual journals” [31].

In this report, we provided evidence suggesting that although the kinetics of journal self-cites is faster compared to foreign cites across multiple research fields, it shows some field-specific characteristics. Interestingly, we found out that among the three research fields analyzed the highest temporal peak of self-citations was associated with *Multidisciplinary sciences* journals. In contrast, the difference between the kinetics of journal self-cites and foreign cites in *Parasitology* journals and in *information science* journals was much lower. Particularly in the *information science* journals, the initial increase in a share of journal self-citations during post-publication year 0 was completely absent (**Fig 1**). This actually corresponded to the annual journal self-citation ratios, which were increased during first two post-publication years in *Multidisciplinary sciences* and *Parasitology* journals but which were nearly absent in most *information science* journals (**Fig 2**). The causes of this difference are unclear. Perhaps the publication mode of the information science journals may contribute to it, as numerous information science journals display long lag periods between manuscript acceptance and publication, and are also published with a limited frequency, which delays the self-citations compared to the weekly or bi-weekly journals characteristic for the top-tier *Multidisciplinary sciences* journals. But part of this difference may be also attributed to a different publication habits of the authors publishing in top-tier *Multidisciplinary science* journals, where most of the space is occupied by novel and only temporarily attractive topics, whereas the *information science* journals included in the analysis consisted of journals selected irrespectively of their impact factor.

The self-promotion of articles published by the same journal or by the same publisher could be considered acceptable. But as some journals adopted and support this strategy, whereas the others do not publish any single self-promoting article, the differences in their behavior affects their citation indices. It would be of interest to know how effective the self-promotional articles actually are in attracting more citations to the primary research they refer to. From my own experience, I think this is an additional, yet overlooked factor, although it is not subject to any controversy. I recall many times when I actually looked for a research article after reading the editorial written by members of the editorial board or one of the reviewers in the same journal issue, and occasionally used and cited it in my own research. So to have the self-promoting articles pays even in the ethical way. Due to similar reasons, many journals encourage (or demand) to submit features such as highlights, graphical abstracts, stand-alone statements regarding clinical significance, audioslides, etc., or highlight selected papers on their web page (as *PLoS ONE* does). It is nearly impossible to track the exact reasons leading to each particular download or citation of the respective paper. However, it is possible to track self-cites, which represent too obvious method (mis-)used to attract attention to the journal. When analyzing two top-tier biomedical journals, we have shown here that actually striking 98.9% of the variability in annual citation counts can be explained by journal self-cites and cites by other journals of the same publisher, and that particularly cites received during post-publication year 0 are affected by this issue (**Fig 3**).

Whereas the skewed pattern of cites by self-cites in top-tier biomedical journals affects only marginally their impact factor, and should probably be treated more as a form of advertisement instead of unethical behavior (as indicated by the dynamics of such self-cites and cites by other journals of the same publisher), in other journals, there occurs also citation stacking. The possibility to arrange citation stacking was mentioned for a first time probably by G. Franck in 1999 [32], who proposed that groups of editors or journals may work together for mutual benefits, although there was no documentation for such behavior at that time. The first broadly medialized case of citation stacking appeared in 2012, when Thomson Reuters reported an exclusion of 51 journals from JCR. Whereas 48 of them were blacklisted due to excessive number of journal self-cites (similarly to the bans announced in previous years, although in lower numbers), three journals were banned due to citation stacking. These three journals–*Cell Transplantation*, *Medical Science Monitor* and *The Scientific World Journal* were alleged to work apparently together to cite each other and thus raise their impact factors [28]. This case included a publication of a single paper in *Medical Science Monitor* citing 445 papers published in *Cell Transplantation* within the impact factor calculation window, and another 44 papers published in *Medical Science Monitor* within the impact factor calculation window (the same paper contained just a single additional citation adding up to total sum of citations equal to 490). One of the four authors of the paper was a founding editor of *Cell Transplantation*, two others were its associate editors. Other similar papers appeared in all the three journals, citing each other in hundreds of citations. Also a fourth journal, *Technology and Innovation*, “benefited” from couple dozens of citations in a similar way [33]. Such praxis may be more widespread than we actually think about. Journal self-citation networks can be formed even by a single person, as pinpointed recently by John P. A. Ioannidis [18]. He noticed that the 14^th^ most prolific author in the field of biomedicine in years 2008–2012 according to his *h* index calculated by Microsoft Academic Search, the Academy-of-Athens-based Dimitrios H. Roukos, received over a thousand of cites from papers thought to be authored by his mentor, who ceased his publication activity in English already in 1994, but restarted “his” publication activity in 2008, publishing numerous letters in journals *Surgical Endoscopy* and *Annals of Surgical Oncology*. The e-mail of this “author” referred to a website launched by Roukos, and the letters typically contained relatively large lists of references, with >80% of references to papers authored by Roukos (but not vice versa) [18]. Alternatively, the letters were published also under names of Greek authors, such as J. Spiliotis, O. Zoras, C. Katsios, C. Batsis, and others, all citing predominantly the papers by Roukos. Whereas the citing papers were published nearly exclusively in *Surgical Endoscopy* and *Annals of Surgical Oncology*, the cited papers by Roukos were published not only in these two journals but also in *Annals of Surgery*, *Gastric and Breast Cancer*, *Expert Review of Anticancer Therapy*, and many others (P. Heneberg, pers. obs.). It is assumed that such citation pattern cannot be established without any support provided by editorial boards of affected citing journals.

Unlike self-citation, which is easy to detect by automated algorithms, Thomson Reuters currently does not have any algorithm to detect citation stacking nor any publicly claimed policy leading to the eradication of such behavior [33]. Here we identified a new case of citation stacking, which was characteristic by the absence of self-cites within post-publication year 0 and with sharp decrease of self-cites with the end of impact factor calculation window. There were three journals involved, all published by a single Romanian publisher. All three of them displayed strong increase of their impact factors (although, their impact factors were still relatively low, they multiplied 2.7–11.1× during the period 2010–2014). All also displayed relatively low to moderate amount of self-cites both within (13.8–33.8%) and outside (10.2–25.6%) the impact factor calculation window (**Fig 5**), which enabled them to escape the JCR policy blacklisting the journals with excessive number of self-cites. Despite of that, self-cites within the network of three journals reached 55.9%, 64.7% and 63.3% in the three journals, respectively. The suspicious character of cites within the network was supported by the fact that there were not nearly any network cites prior the impact factor calculation window, and they also decreased sharply after the impact factor calculation window.

Concluded, currently used scientometric indicators provide only limited protection against the unethical behavior discussed in this article, and are also prone to be shifted due to perhaps unintentional behavior aimed to advertise the primary research published by the journal or a network of journals. An algorithm is needed to be developed to search for potential citation networks, allowing their efficient elimination. The algorithm could be based on differences in a number of citations received from a respective journal during the impact factor calculation window (post-publication years 1–2) and the number of citations received only later (e.g., post-publication years 4–7). The self-promoting journal self-citations (such as those employed by publishers American Association for the Advancement of Science or Nature Publishing Group) have rather indirect effects. They have negligible direct influence on impact factor calculation, affecting just the immediacy index and marginally increasing the impact factor itself as long as the affected journals are well established in their fields. In contrast, other forms of journal self-citations and citation stacking may severely affect the impact factor itself (or any citation-based score, which the journal editorial board would consider important), and thus require increased attention and elimination from citation indices.

## Supporting Information

S1 Table The journals analyzed, their ISSN and IF_2014_ according to the Journal Citation Reports 2014 edition. (DOCX)
