# Supplementary material for: From Excessive Journal Self-Cites to Citation Stacking: Analysis of Journal Self-Citation Kinetics in Search for Journals, Which Boost Their Scientometric Indicators
Source: PLoS One. 2016 Apr 18;11(4):e0153730. doi: 10.1371/journal.pone.0153730 (PMC4835057; doi:10.1371/journal.pone.0153730)
Supplement: S1 Table — (DOCX) [file pone.0153730.s001.docx]

*Multidisciplinary sciences* journals analyzed in this study (top 20 according to IF_2014_)

| \| Rank \| \| --- \| | Journal Title | ISSN | IF_2014_ |
| --- | --- | --- | --- | --- |
| 1 | NATURE | 0028-0836 | 41.456 |
| 2 | SCIENCE | 0036-8075 | 33.611 |
| 3 | NAT COMMUN | 2041-1723 | 11.470 |
| 4 | P NATL ACAD SCI USA | 0027-8424 | 9.674 |
| 5 | SCI REP-UK | 2045-2322 | 5.578 |
| 6 | ANN NY ACAD SCI | 0077-8923 | 4.383 |
| 7 | J R SOC INTERFACE | 1742-5689 | 3.917 |
| 8 | RES SYNTH METHODS | 1759-2879 | 3.898 |
| 9 | PLOS ONE | 1932-6203 | 3.234 |
| 10 | P JPN ACAD B-PHYS | 0386-2208 | 2.652 |
| 11 | P ROY SOC A-MATH PHY | 1364-5021 | 2.192 |
| 12 | PHILOS T R SOC A | 1364-503X | 2.147 |
| 13 | PEERJ | 2167-8359 | 2.112 |
| 14 | NATURWISSENSCHAFTEN | 0028-1042 | 2.098 |
| 15 | P ROMANIAN ACAD A | 1454-9069 | 1.658 |
| 16 | CHINESE SCI BULL | 1001-6538 | 1.579 |
| 17 | JOVE-J VIS EXP | 1940-087X | 1.325 |
| 18 | FRACTALS | 0218-348X | 1.220 |
| 19 | INT J BIFURCAT CHAOS | 0218-1274 | 1.078 |
| 20 | SCI AM | 0036-8733 | 1.070 |

*Parasitology* journals analyzed in this study (top 20 according to IF_2014_)

| \| Rank \| \| --- \| | Journal Title | ISSN | IF_2014_ |
| --- | --- | --- | --- | --- |
| \| 1 \| \| --- \| | CELL HOST MICROBE | 1931-3128 | 12.328 |
| 2 | PLOS PATHOG | 1553-7366 | 7.562 |
| 3 | ADV PARASIT | 0065-308X | 6.226 |
| 4 | TRENDS PARASITOL | 1471-4922 | 6.204 |
| 5 | PLOS NEGLECT TROP D | 1935-2735 | 4.446 |
| 6 | INT J PARASITOL | 0020-7519 | 3.872 |
| 7 | PARASITE VECTOR | 1756-3305 | 3.430 |
| 8 | INT J PARASITOL-DRUG | 2211-3207 | 3.294 |
| 9 | MALARIA J | 1475-2875 | 3.109 |
| 10 | TICKS TICK-BORNE DIS | 1877-959X | 2.718 |
| 11 | PARASITOLOGY | 0031-1820 | 2.560 |
| 12 | VET PARASITOL | 0304-4017 | 2.460 |
| 13 | ACTA TROP | 0001-706X | 2.270 |
| 14 | PARASITE IMMUNOL | 0141-9838 | 2.143 |
| 15 | PARASITOL RES | 0932-0113 | 2.098 |
| 16 | PARASITOL INT | 1383-5769 | 1.859 |
| 17 | MOL BIOCHEM PARASIT | 0166-6851 | 1.787 |
| 18 | PATHOG GLOB HEALTH | 2047-7724 | 1.656 |
| 19 | EXP PARASITOL | 0014-4894 | 1.638 |
| 20 | MEM I OSWALDO CRUZ | 0074-0276 | 1.592 |

*Scientometrics* journals analyzed in this study [top 20 according to WoS search TOPIC: ("scientometric*" or "bibliometric*" or "informetric*" or "citation*"), refined by RESEARCH AREAS: (INFORMATION SCIENCE LIBRARY SCIENCE or SCIENCE TECHNOLOGY OTHER TOPICS or COMPUTER SCIENCE OR SOCIAL SCIENCES OTHER TOPICS). The search revealed 16,399 results, which, however, contained still numerous multidisciplinary journals, proceedings and technologically-oriented journals. These were manually removed, and the first 20 journals according to the number of matching papers were listed below, sorted according to the number of matching papers]

| \| Rank \| \| --- \| | Journal Title | ISSN | IF_2014_ |
| --- | --- | --- | --- | --- |
| 1 | SCIENTOMETRICS | 0138-9130 | 2.183 |
| 2 | J AM SOC INF SCI TEC | 1532-2882 | 1.846 |
| 3 | J INFORMETR | 1751-1577 | 2.412 |
| 4 | J INF SCI | 0165-5515 | 1.158 |
| 5 | RES EVALUAT | 0958-2029 | 1.123 |
| 6 | J ASSOC INF SCI TECH | 2330-1635 | N/A |
| 7 | REV ESP DOC CIENT | 0210-0614 | 0.636 |
| 8 | LIBR INFORM SCI RES | 0740-8188 | 1.153 |
| 9 | ONLINE INFORM REV | 1468-4527 | 0.918 |
| 10 | MALAYS J LIBR INF SCI | 1394-6234 | 0.238 |
| 11 | LIBR RESOUR TECH SER | 0024-2527 | 0.452 |
| 12 | INVESTIG BIBLIOTECOL | 0187-358X | 0.104 |
| 13 | J ACAD LIBR | 0099-1333 | 0.448 |
| 14 | ASLIB PROC | 0001-253X | 0.676 |
| 15 | PROF INFORM | 1386-6710 | 0.356 |
| 16 | J AM MED INFORM ASSN | 1067-5027 | 3.504 |
| 17 | LIBR TRENDS | 0024-2594 | 0.386 |
| 18 | LIBR QUART | 0024-2519 | 0.500 |
| 19 | ELECTRON LIBR | 0264-0473 | 0.535 |
| 20 | CAN J INFORM LIB SCI | 1195-096X | 0.167 |

*Editura Academiei Române* physics journals analyzed in this study

| \| Rank \| \| --- \| | Journal Title | ISSN | IF_2014_ |
| --- | --- | --- | --- | --- |
| \| 1 \| \| --- \| | P ROMANIAN ACAD A | 1454-9069 | 1.658 |
| 2 | ROM REP PHYS | 1221-1451 | 1.517 |
| 3 | ROM J PHYS | 1221-146X | 0.924 |

*Nature*, *Nature series*, *Science* and *Science Signaling* journals analyzed in this study (sorted alphabetically)

| \| Rank \| \| --- \| | Journal Title | ISSN | IF_2014_ |
| --- | --- | --- | --- | --- |
| \| 1 \| \| --- \| | NATURE | 0028-0836 | 41.456 |
| 2 | NAT BIOTECHNOL | 1087-0156 | 41.514 |
| 3 | NAT CELL BIOL | 1465-7392 | 19.679 |
| 4 | NAT CHEM BIOL | 1552-4450 | 12.996 |
| 5 | NAT CHEM | 1755-4330 | 25.325 |
| 6 | NAT CLIM CHANGE | 1758-678X | 14.547 |
| 7 | NAT COMMUN | 2041-1723 | 11.470 |
| 8 | NAT GENET | 1061-4036 | 29.352 |
| 9 | NAT GEOSCI | 1752-0894 | 11.740 |
| 10 | NAT IMMUNOL | 1529-2908 | 20.004 |
| 11 | NAT MATER | 1476-1122 | 36.503 |
| 12 | NAT MED | 1078-8956 | 28.223 |
| 13 | NAT METHODS | 1548-7091 | 32.072 |
| 14 | NAT NANOTECHNOL | 1748-3387 | 34.048 |
| 15 | NAT NEUROSCI | 1097-6256 | 16.095 |
| 16 | NAT PHOTONICS | 1749-4885 | 32.386 |
| 17 | NAT PHYS | 1745-2473 | 20.147 |
| 18 | NAT PROTOC | 1754-2189 | 9.673 |
| 19 | NAT REV CANCER | 1474-175X | 37.400 |
| 20 | NAT REV CARDIOL | 1759-5002 | 9.183 |
| 21 | NAT REV CLIN ONCOL | 1759-4774 | 14.180 |
| 22 | NAT REV DRUG DISCOV | 1474-1776 | 41.908 |
| 23 | NAT REV ENDOCRINOL | 1759-5029 | 13.281 |
| 24 | NAT REV GASTRO HEPAT | 1759-5045 | 12.610 |
| 25 | NAT REV GENET | 1471-0056 | 36.978 |
| 26 | NAT REV IMMUNOL | 1474-1733 | 34.985 |
| 27 | NAT REV MICROBIOL | 1740-1526 | 23.574 |
| 28 | NAT REV MOL CELL BIO | 1471-0072 | 37.806 |
| 29 | NAT REV NEPHROL | 1759-5061 | 8.542 |
| 30 | NAT REV NEUROL | 1759-4758 | 15.358 |
| 31 | NAT REV NEUROSCI | 1471-003X | 31.427 |
| 32 | NAT REV RHEUMATOL | 1759-4790 | 9.845 |
| 33 | NAT REV UROL | 1759-4812 | 4.840 |
| 34 | NAT STRUCT MOL BIOL | 1545-9993 | 13.309 |
| 35 | SCIENCE | 0036-8075 | 33.611 |
| 36 | SCI SIGNAL | 1945-0877 | 6.279 |
